# Supplementary material for: Pseudomonas aeruginosa alkyl quinolone response is dampened by Enterococcus faecalis
Source: mBio. 2024 Dec 27;16(2):e03320-24. doi: 10.1128/mbio.03320-24 (PMC11796420; doi:10.1128/mbio.03320-24)
Supplement: Supplemental Material — Supplemental methods, Tables S1 to S3, and Fig. S1 to S8. [file mbio.03320-24-s0001.pdf]

SUPPLEMENTARY INFORMATION

Pseudomonas aeruginosa Alkyl Quinolone Response is dampened by *Enterococcus faecalis*

Maggie M. Fink[a], Abigail A. Weaver[b], Dharmeshkumar Parmar[c], Jon E. Paczkowski[d,e], Lingyun Li[f], Maggie K. Klaers[a] Ella A. Junker[b], Elizabeth A. Jarocki[b], Jonathan V. Sweedler[c], Joshua D. Shrout[a,b#]

[a]Department of Biological Sciences, University of Notre Dame, Notre Dame, Indiana 46556, USA

[b]Department of Civil and Environmental Engineering and Earth Sciences, University of Notre Dame, Notre Dame, Indiana 46556, USA

[c]Department of Chemistry and Beckman Institute for Advanced Science and Technology, University of Illinois at Urbana-Champaign, Urbana, Illinois 61801, USA

[d]Department of Biomedical Sciences, University at Albany, School of Public Health, Albany, New York, 12201, USA.

[e]Division of Genetics, Wadsworth Center, New York State Department of Health, Albany, New York, 12208, USA.

[f]Division of Environmental Health Sciences, Wadsworth Center, New York State Department of Health, Albany, New York, 12208, USA.

[#]corresponding author, email: joshua.shrout@nd.edu

Supplemental Methods

Mass Spectrometry Imaging Analysis

Samples for MALDI-MSI analysis were prepared by cutting and removing a center square section of agar from the petri dish, affixing this section to aluminum plates using copper tape, and dehydrating overnight under forced air (1). These dehydrated agar samples were uniformly coated with 2,5-dihydroxy benzoic acid (DHB) (40 mg/mL in 50% Methanol) using M5 overhead matrix sprayer (HTX, USA). The spray parameters were maintained as follows, nozzle height - 40 mm, nozzle temperature - 70 ˚C, lateral nozzle speed - 1000 mm, lateral spacing - 3 mm, matrix flow rate - 0.05 mL/min and nitrogen pressure - 1 bar. Sample surface was coated with DHB in 4 passes with each layer sprayed at 90˚ to the previous one and 10 s drying time between passes. The plates were stored inside the nitrogen desiccator until analysis.

Mass spectrometry imaging was performed on FT-ICR mass spectrometer (solariX 7T, Bruker, USA) equipped with matrix-assisted laser desorption/ionization (MALDI) source. To facilitate MSI, the samples were scanned using a flatbed document scanner at 1200 DPI. Regions of interest for MSI were selected in FlexImaging (version 5.0, build 89). The MS parameters were set using ftmsControl (version 2.3.0, build 59). The data acquisition was carried out in the positive ion mode with laser size set at “ultra-large”, and MS analyzer operated at 1M size (transient data points). Targeted MS/MS (collision energy 30 eV, isolation width ±1.0 Da) was performed to differentiate PQS (*m/z* 260.165>175.062) and its structural isomer HQNO *(m/z* 260.165>159.067), as well as C9-PQS (*m/z* 288.195>175.062) and NQNO (*m/z* 288.195>159.067). Separately, imaging data in a broad mass range (*m/z* 100-1600) was acquired to detect other

metabolites, such as pyocyanin. The MSI data was processed in the SCILS lab (Version 2023a Pro). Extracted ion profiles were generated using ±16 ppm extraction window around the observed *m/z*. Extracted ion intensities have been represented using false fire color.

Normalized intensity for analyzed regions were calculated as: {Average intensity from the inoculation zone *second species* }/{Average intensity from the sampling region}.

The normalized average intensity value of 1 (y-axis) indicates the average level in the sampling region. The sampling region is the whole area and includes the intensities from *P. aeruginosa* zone. Normalized average intensity values <1 indicate a relative decrease in the metabolite abundance, and values >1 indicate an increase in the abundance.

Reporter Strain Construction

Standard genetic techniques were used to construct chromosomal (Tn7) transcriptional reporter strains to be used as indicators of *P. aeruginosa* AQ or AHL quorum sensing pathway activity. Strains, plasmids, and DNA sequences are included in Tables S1, S2 and S3, respectively. All sequences were designed starting with the PAO1 sequence as a reference strain as annotated on pseudomonas.com (2). A transcriptional reporter for *pqsH* was generated by first amplifying *P. aeruginosa* chromosomal DNA roughly 560 bp upstream of *pqsH* utilizing using the primer pairs listed in Table S3. All primers and gBlocks were obtained from IDT. PCR reactions utilized AllTaq Master Mix (Qiagen) and products cloned with super-folder green

fluorescent protein (sfGFP) by isothermal assembly into pUC18-mini-Tn7T-Gm using NEBuilder HiFi Assembly Master Mix and transformed into *E. coli* DH5α (New England Biolabs).

A transcriptional short-half life reporter for *hcnA* was generated by first amplifying *P. aeruginosa* chromosomal DNA previously assessed as regulating transcription of *hcnABC* in *P. fluorescens* (3) using primers in Table S3. A 280 bp fragment was generated by restricting with EcoRI and XbaI, which was cloned into AvrII and EcoRI restricted DNA of pJDS5 to create pJDS116.

Plasmid DNA was transformed by heat-shock into *E. coli* SM10 cells. Conjugational mating was used promote uptake of Tn7 transposons into *P. aeruginosa* (4). Colonies were screened on LB plates supplemented with 100 μg/ml gentamicin or 100 μg/ml tetracycline.

Fluorescence microscopy of Reporter Strains.

P. aeruginosa harboring chromosomally inserted P_{pqsH}-gfp, P_{rhlA}-gfp, P_{hcnA}-gfp and P_{rsaL}-gfp transcriptional fluorescent reporters were imaged to judge the impact of *E. faecalis*, *E. coli*, or exogenous ornithine on relative levels of gene expression. Some of these assays utilized an *E. faecalis* Δ*arcD* mutant (Table S1) that is derived from the *E. faecalis* OG1RF background. (We find the OG1RF wildtype to have equivalent growth to that of EfPJI-A5; c.f. Figure 1 and Figure S8). Images were obtained using a Leica DM6B upright microscope equipped with a 10× Fluotar objective with simultaneous excitation at 475 nm with emission capture using settings of 525 ± 50 nm. Grey scale images were obtained using brightfield. From each region of interest, ten replicate

sections (48 × 50 pixels) were sampled to determine the integrated fluorescence density as measured using ImageJ (5).

Quantification of reporter expression for planktonic cultures over time using a microplate reader (Biotek, Synergy H1). *P. aeruginosa* wildtype harboring P_{pqsH}-gfp, P_{rhlA}-gfp, P_{hcnA}-gfp or P_{rsaL}-gfp reporter constructs was added to 200 µL FAB-glucose in black 96-well clear bottom plates (Agilent) to an initial OD of 0.01. Absorbance at 600 nm and fluorescence at excitation of 488 nm with emission capture at 525 nm were measured every 10 minutes for 24 hours at 37°C. The plate was shaken for 10 seconds before each read.

E. faecalis monoculture with P. aeruginosa supernatant growth conditions

The *P. aeruginosa* wildtype and Δ*pqsA* strains were each grown planktonically in 6mL FAB medium supplemented with 12 mM glucose while *E. faecalis* EfPJI-A5 was grown planktonically in Luria-Bertani (LB) broth. These planktonic cultures were incubated for 20 hours at 37°C with shaking at 240 rpm. Spent-culture supernatant was harvested from the *P. aeruginosa* cultures was by filter-sterilizing the resultant supernantant after 10 minutes centrifugation.

Using clear 96-well plates, 100 µL of *P. aeruginosa* wildtype or Δ*pqsA* spent culture supernatant was added to 100 µL sterile FAB-glucose. Non-supernatant controls contained 100 µL sterile FAB. Wells were then inoculated with 5 µL *E. faecalis* that was diluted 1 in 5 from the pre-grown planktonic culture. The plate was grown at 37°C with 10 seconds shaking every 10 minutes prior to each optical density measurement at 600 nm using a microplate reader (Biotek, Synergy H1).

Pyocyanin Quantification

Pyocyanin quantification was adapted from Frank and Demoss (6). To extract pyocyanin from planktonic cultures, 6 mL planktonic cultures were grown for 24 hours, then centrifuged at 12,000 rpm for 20 minutes at 4°C. Supernatants were collected, filtered with 0.22µm sterile filters, and mixed with 3 mL of chloroform. After vortexing for 20 seconds and allowing the aqueous and organic phases to separate, the top aqueous layer was removed. 20% total volume of 0.1N HCL was added to the chloroform fraction containing the pyocyanin and allowed to settle for 10 minutes. The aqueous solution containing crude pyocyanin extract was removed and absorbance was measured at 520 nm in a black 96-well glass bottom plate (Aligent) using a microplate reader (Biotek, Synergy H1).

Ornithine quantification

Monocultures and cocultures were grown as described for determining CFUs. At 24h cultures were spun down at 10,000 rpm for 20 minutes and supernatant was filtered using 0.22 µm PES syringe filters (Avantor). A hybrid Hydrophilic Interaction Liquid Chromatography (HILIC) coupled with a high-resolution mass spectrometer (HRMS) method was used for ornithine quantitative analysis. A high-throughput and direct sample extraction method was developed for cell-free supernatant sample analysis. Briefly, 100 µL of filtered supernatant samples were directly extracted using 900 µL acetonitrile/water mixture (9:1) and vortexed for 15 s. The precipitated proteins were separated from the extraction solvent using centrifugation (2 min, 10,000 × g). The

supernatant was directly used for HILIC-HRMS analysis. For HILIC-HRMS high-throughput analysis, the instrumental system included a Vanquish liquid chromatography system coupled with a high-resolution QE Orbitrap mass spectrometer (ThermoFisher) operating in the positive electrospray-ionization (ESI) mode with a heated ion source (HESI) running at parallel reaction monitoring (RPM) mode. Analyte separation was achieved using a HILIC column (Raptor Polar X, 2.7 µm, 100 mm x 2.1 mm, Restek) as the stationary phase and solvent A and B as mobile phases. For gradient elution, mobile phase A was 0.5% formic acid, 1 mM ammonium formate in water, and mobile phase B was 0.5% formic acid, 1 mM ammonium formate in acetonitrile: water (90:10). The flow rate was 0.40mL/min, and the column was maintained at 35 °C. The binary gradient was initially at 96% mobile phase B and decreased to 15% mobile phase B over 4.5 min. After a hold at 5% mobile phase B from 4.6-5.4 min, the mobile phase composition was returned to 96% mobile phase B in 0.1min and maintained for another 2 min to equilibrate the column. The sample injection volume was 2 µL. An external calibration curve was used for quantifying the ornithine in the samples with quality control samples to ensure the system stability was qualified for each batch. We used a standard spike-recovery rate in this analysis. For quantification, a 5 parts per million (ppm) accurate mass window was used for peak integration of the high-resolution MS/MS data acquired using the PRM method. The limit of quantification of 0.30 µM ornithine was achieved using this method. Data were acquired and processed with TraceFinder 5.0 software (ThermoFisher).

Table S1: Strains used in this study

Strain	Select Characteristics	Reference
Escherichia coli		
DH5α	High-efficiency for cloning	New England Biolabs
SM10	Conjugational competent donor; λpir	(7)
K12	Standard wild-type strain	(8)
Pseudomonas aeruginosa		
PAO1C	Wildtype Laboratory Strain, ATCC Collection Strain 15692	(9-12)
Δ*pqsA*	PAO1C Δ*pqsA*	(13)
wt-P*rhlA*-gfp	15692 P_{rhlA}-*gfp* reporter; Tc^r	(14)
wt-P*rsaL*-gfp	15692 P_{rsaL}-*gfp*(AAV) reporter; Tc^r	(15)
wt-P*hcnA*-gfp	15692 P_{hcnA}-*gfp*(AAV) reporter; Tc^r	This study
wt-P*pqsH*-gfp	15692 P_{pqsA}-gfp reporter; Gm^r	This study
Enterococcus faecalis		
EfPJI-A5	Clinical isolate from prosthetic joint infection	(16)
10103	OG1RF Δ*arcD*	(17)
Staphylococcus aureus		
SaPJI-C	Clinical isolate from prosthetic joint infection	(16)
Clostridium striatum		
CsPJI-A24	Clinical isolate from prosthetic joint infection	(16)

Table S2: Plasmids used in this study

Plasmid	Select Characteristics	Reference
pUC18-mini-Tn7T-Gm	Ap^r; Gm^r on mini Tn7T	(18, 19)
pTNS2	Ap^r; R6K replicon; TnsABC+D transposition pathway	(18, 19)
pJDS5	p-miniCTX-P_{rsaL}::*gfp*(ASV); Tc^r	(15)
pJDS116	p-miniCTX-P_{hcnA}::*gfp*(ASV); Tc^r	This study
pJDS151	pUC18-mini-Tn7T-Gm-P_{pqsH}-sfgfp	This study

Table S3: Primers and DNA used in this study

**Primer**	**Sequence**
PpqsH_EcoRI_UP-F	GATCCCCCGGGCTGCAGGAATTCTTCAGCACGATCCACTCGTAG
PpqsH_sfGFP_UP-R	TCTTCTCCTTTGCTCATCCGTTGCTCCTTAGCAGCGGCATC
EcoRI-PhcnA-F	GAATTCCGTCGCTGTCGGTGAACGAA
XbaI-PhcnA-R	TCTAGATTGCCCTTTCATCCGTGAGA
PpqsH-sfGFP_DN-F	GCTAAGGAGCAACGGATGAGCAAAGGAGAAGAACTTTT
sfGFP_HindIII_DN-R	CGCGAGGTACCGGGCCCAAGCTTTTACGCTGCAAGGGCGTAATTTTC
sfgfp-F	ATGAGCAAAGGAGAAGAACTTTT
sfgfp-R	TACGCTGCAAGGGCGTAATTTTCG
sfgfp gblock	ATGAGCAAAGGAGAAGAACTTTTCACTGGAGTTGTCCCAATTCTT<br>GTTGAATTAGATGGTGATGTTAATGGGCACAAATTTTCTGTCCGT<br>GGAGAGGGTGAAGGTGATGCTACAAACGGAAAACTCACCCTTAA<br>ATTTATTTGCACTACTGGAAAACTACCTGTTCCGTGGCCAACACT<br>TGTCACTACTCTGACCTATGGTGTTCAATGCTTTTCCCGTTATCC<br>GGATCACATGAAACGGCATGACTTTTTCAAGAGTGCCATGCCCG<br>AAGGTTATGTACAGGAACGCACTATATCTTTCAAAGATGACGGG<br>ACCTACAAGACGCGTGCTGAAGTCAAGTTTGAAGGTGATACCCT<br>TGTTAATCGTATCGAGTTAAAGGGTATTGATTTTAAAGAAGATGG<br>AAACATTCTTGGACACAAACTCGAGTACAACTTTAACTCACACAA<br>TGTATACATCACGGCAGACAAACAAAAGAATGGAATCAAAGCTAA<br>CTTCAAAATTCGCCACAACGTTGAAGATGGTTCCGTTCAACTAGC<br>AGACCATTATCAACAAAATACTCCAATTGGCGATGGCCCTGTCCT<br>TTTACCAGACAACCATTACCTGTCGACACAATCTGTCCTTTCGAA<br>AGATCCCAACGAAAAGCGTGACCACATGGTCCTTCTTGAGTTTG<br>TAACTGCTGCTGGGATTACACATGGCATGGATGAGCTCTACAAA<br>GCAGCGAACGACGAAAATTACGCCCTTGCAGCGTAA

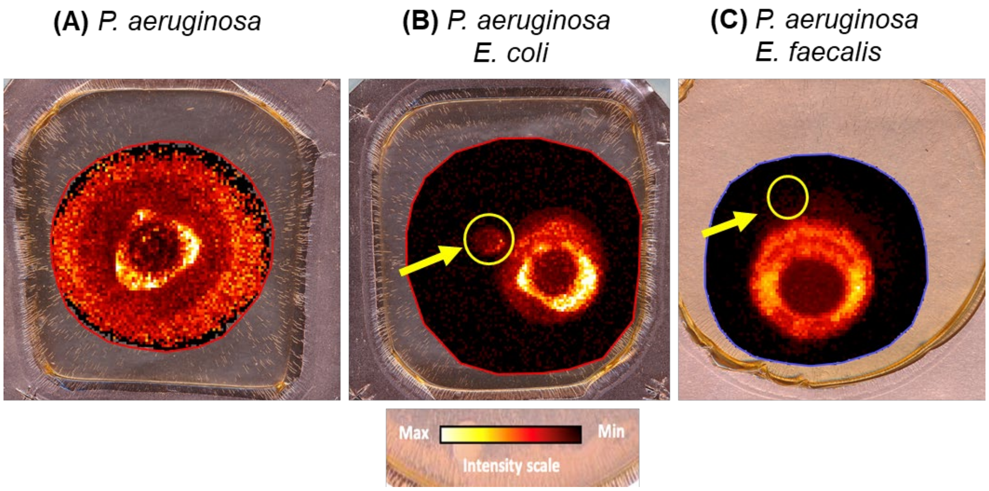

Figure S1. Spatial MSI intensity profiles for PQS show distinct localization patterns for 48-hour colony biofilms of **(A)** *P aeruginosa* alone, near **(B)** *E. coli,* and near **(C)** *E. faecalis.* Images show the PQS intensity heatmap overlayed on the camera image of the agar assay. Each assay shows spatial variability of *P. aeruginosa* produced PQS throughout the colony biofilm. The highest levels appear as a ring surrounding the center of inoculation. When *P. aeruginosa* is co-cultured adjacent to *E. coli* (yellow circle), PQS is detected into the region where *E. coli* has grown. When *P. aeruginosa* is co-cultured adjacent to *E. faecalis* (yellow circle), no apparent increase in PQS is observed in this region.

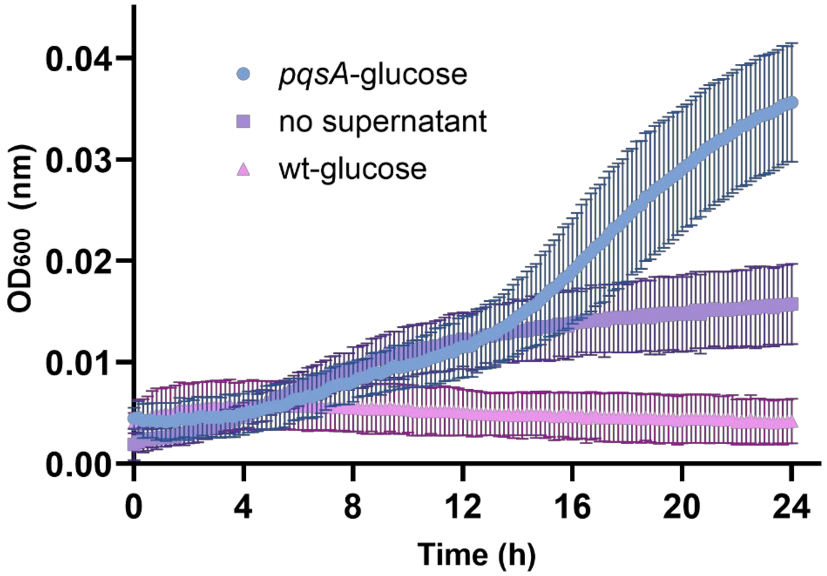

Figure S2. Absence of the *P. aeruginosa* PQS synthesis cascade (Δ*pqsA*) enables *E. faecalis* monoculture growth. Addition of filtered supernatant from spent *P. aeruginosa* Δ*pqsA* enables while *E. faecalis* growth while growth is inhibited with spent *P. aeruginosa* wildtype supernantant. A control condition with unamended medium allows marginal growth of *E. faecalis.* Error bars show standard deviation from ≥8 biological replicates.

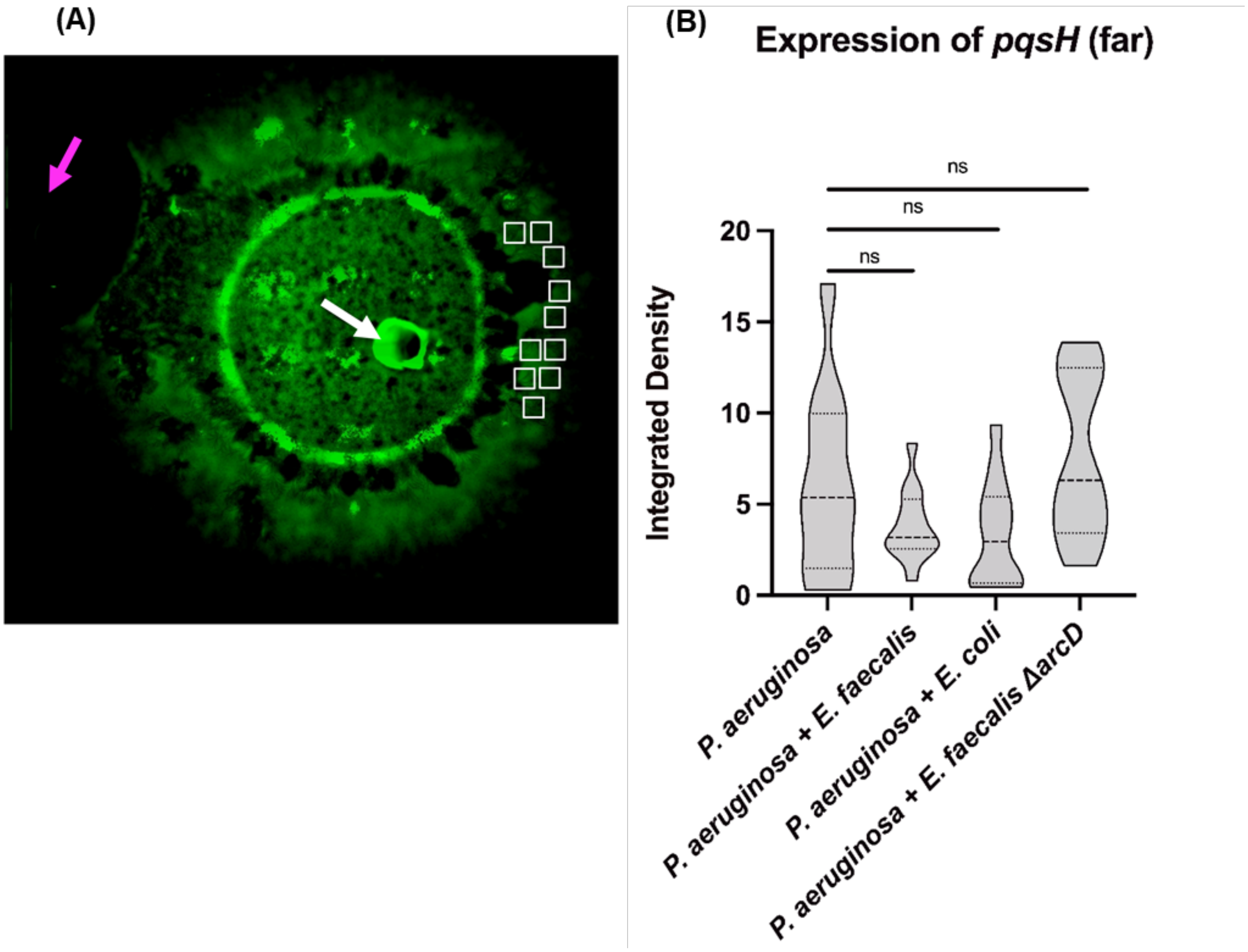

Figure S3. Expression of $P_{pqsH\text{-}sfgfp}$ was measured in *P. aeruginosa* biofilm edge opposite the area of contact with *E. coli* or *E. faecalis.* (This is directly comparable with results shown in Figure 3 that show expression directly adjacent to *E. faecalis*.) **A.** The representative assay shows green fluorescence of $P_{pqsH\text{-}sfgfp}$ by *P. aeruginosa* (inoculated at the white arrow) when *E. faecalis* (inoculated at the pink arrow) is adjacent on the left with the "opposite region". **B.** Integrated densities were acquired from 10 sample areas (white rectangles) on each plate for three biological replicates each for which results were statistically different by one-way ANOVA ($P \leq 0.0001$). Pairwise comparisons by Welch's t-test are indicated on the plot: ns= $P > 0.05$.

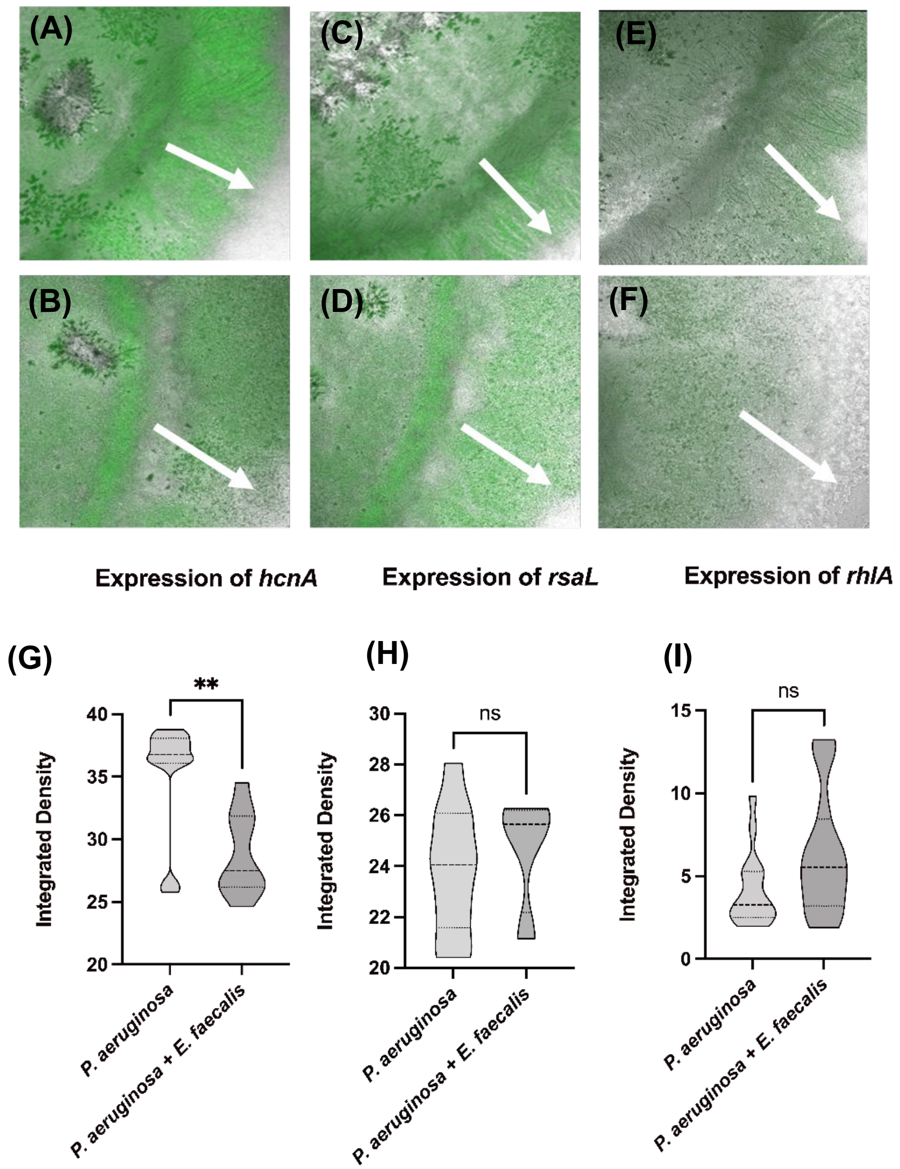

Figure S4. A-F. AHL quorum sensing gene expression is unaffected in proximity to *E. faecalis.* Fluorescence expression of $P_{hcnA\text{-}gfp}$ by *P. aeruginosa* near colony edge is equivalent in surface growth assays for **(A)** *P.a*. monoculture or **(B)** near *E. faecalis*. Expression of $P_{rsaL\text{-}gfp}$ by **(C)** P.a. monoculture or near **(D)** *E. faecalis.* Expression of $P_{rhlA\text{-}gfp}$ by **(E)** *P.a*. monoculture or **(F)** near *E. faecalis*. (The edge of each *P. aeruginosa* colony towards *E. faecalis* is marked by the white arrow, while the *E. faecalis* colony is out of frame.) **G-I.** Quantification of fluorescence corresponding to expression of respective genes. Each reporter strain condition was replicated on three separate plates and comparisons by Welch's t-test are indicated on the plot: ns= P > 0.05, **= P ≤ 0.01.

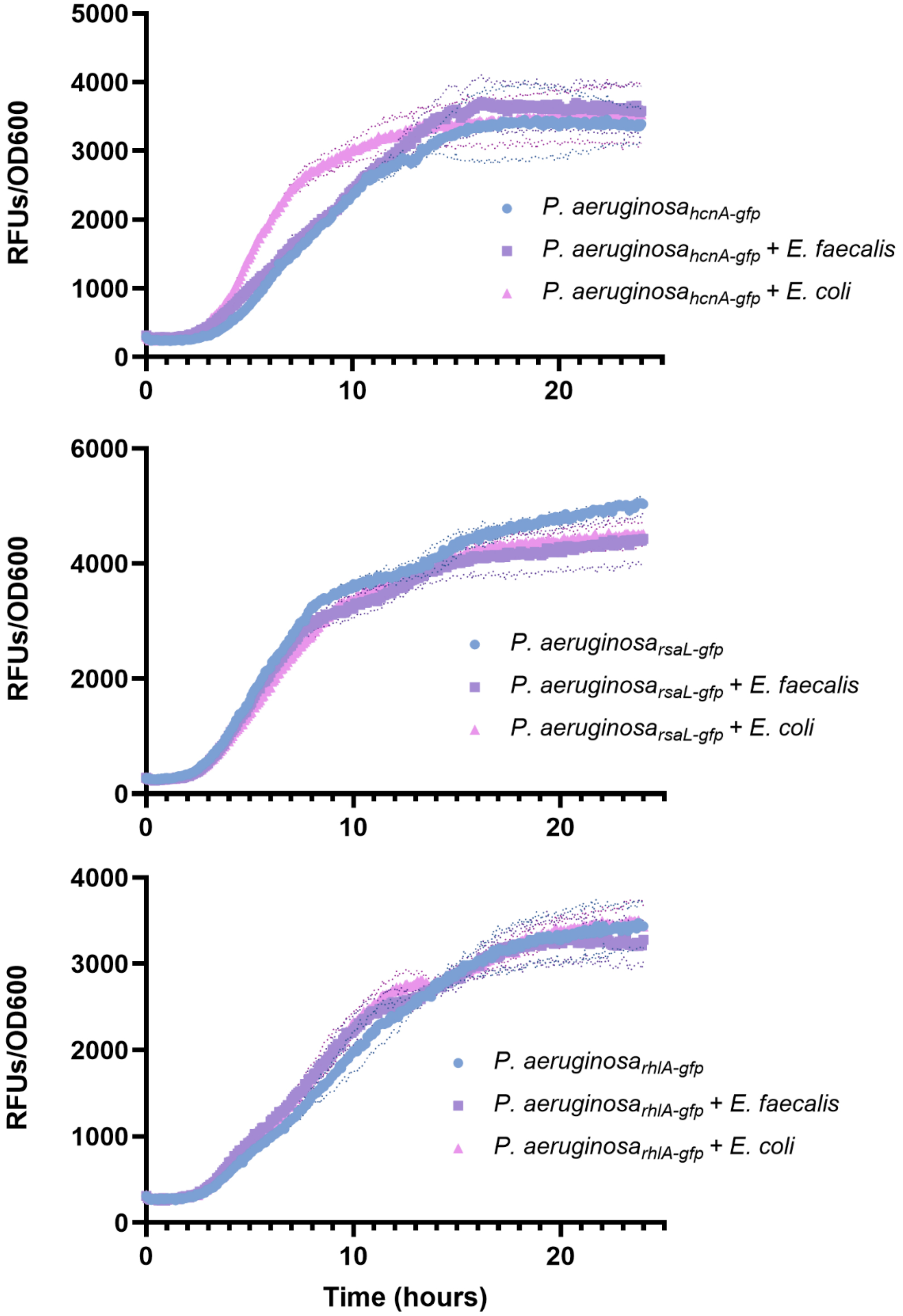

Figure S5. AHL quorum sensing gene expression is unaffected in planktonic co-culture with *E. faecalis.* Fluorescence expression of (A) $P_{hcnA\text{-}gfp}$ (B) $P_{rsaL\text{-}gfp}$ (C) $P_{rhlA\text{-}gfp}$ by *P. aeruginosa* is normalized to culture density over time.

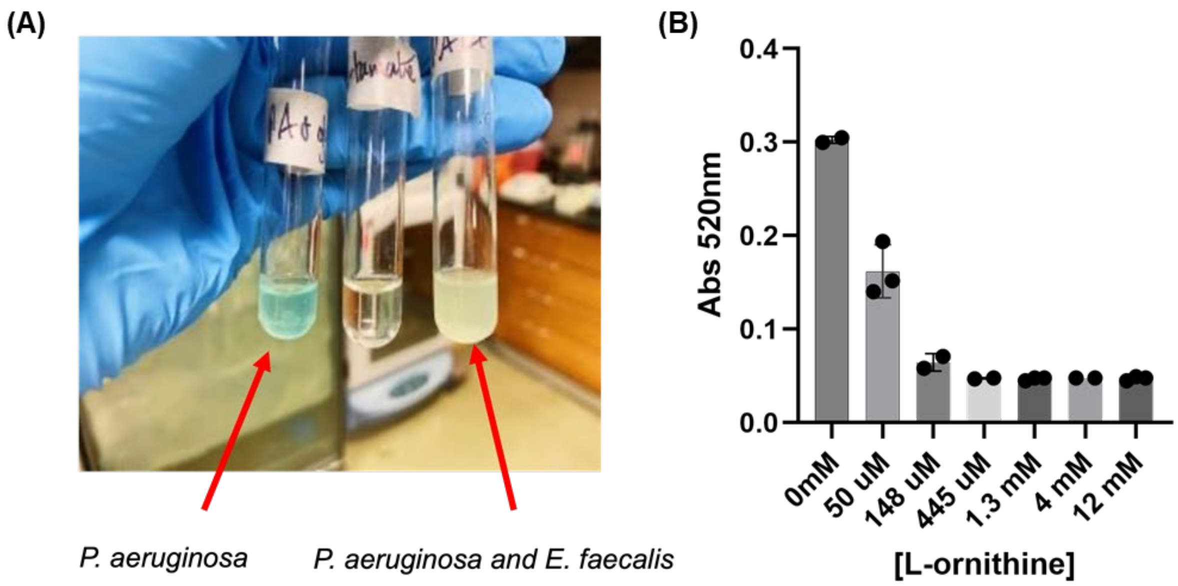

Figure S6. A. The characteristic blue pigment of pyocyanin is present in *P. aeruginosa* monoculture but is absent in co-culture with *E. faecalis.* **B.** Exogenous addition of increasing concentrations of L-ornithine to *P. aeruginosa* cultures decreases pyocyanin production (by Frank and Demoss method (6)). Error bars show ± one standard deviation.

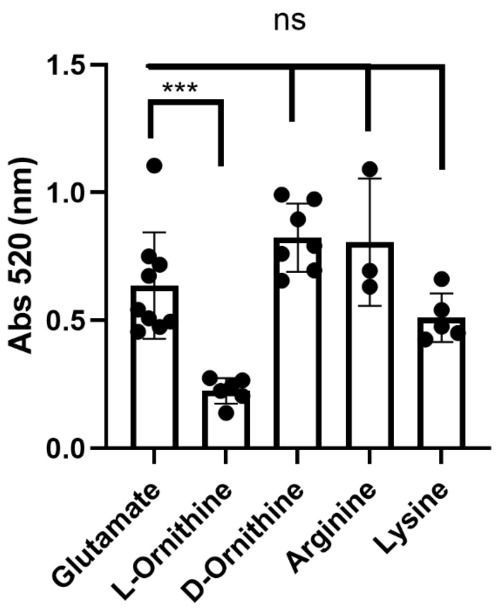

Figure S7. Pyocyanin production is not changed by D-ornithine or other metabolically and structurally related amino acids arginine and lysine. Error bars show standard deviation of ≥3 biological replicates for which results were statistically different by one-way ANOVA ($P \leq 0.0001$). Pairwise comparisons by Welch's t-test are indicated on the plot: ns= $P > 0.05$, ***= $P \leq 0.001$.

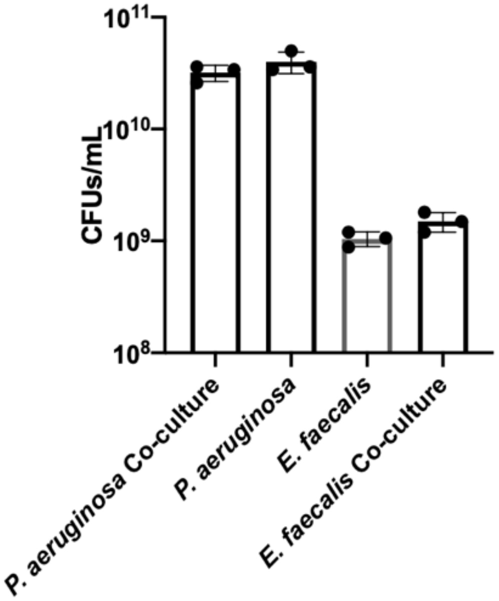

Figure S8. Colony forming units (CFUs) for *P. aeruginosa* and *E. faecalis* OG1RF from monoculture and coculture experiments after 24 hours in Mueller Hinton broth. CFUs were determined from 3 replicates dilutions. Error bars show ± one standard deviation.

Supplemental References

1. **Dunham SJB, Ellis JF, Baig NF, Morales-Soto N, Cao T, Shrout JD, Bohn PW, Sweedler JV.** 2018. Quantitative SIMS Imaging of Agar-Based Microbial Communities Analytical Chemistry.
2. **Winsor GL, Griffiths EJ, Lo R, Dhillon BK, Shay JA, Brinkman FS.** 2016. Enhanced annotations and features for comparing thousands of Pseudomonas genomes in the Pseudomonas genome database. Nucleic Acids Res **44:**D646-653.
3. **Laville J, Blumer C, Von Schroetter C, Gaia V, Defago G, Keel C, Haas D.** 1998. Characterization of the *hcnABC* gene cluster encoding hydrogen cyanide synthase and anaerobic regulation by ANR in the strictly aerobic biocontrol agent *Pseudomonas fluorescens* CHA0. J Bacteriol **180:**3187-3196.
4. **Hoang TT, Kutchma AJ, Becher A, Schweizer HP.** 2000. Integration-proficient plasmids for *Pseudomonas aeruginosa*: Site-specific integration and use for engineering of reporter and expression strains. Plasmid **43:**59-72.
5. **Schneider CA, Rasband WS, Eliceiri KW.** 2012. NIH Image to ImageJ: 25 years of image analysis. Nat Methods **9:**671-675.
6. **Frank LH, Demoss RD.** 1959. On the biosynthesis of pyocyanine. J Bacteriol **77:**776-782.
7. **Simon R, Priefer U, Pühler A.** 1983. A Broad Host Range Mobilization System for In Vivo Genetic Engineering: Transposon Mutagenesis in Gram Negative Bacteria. Bio/Technology **1:**784-791.
8. **Lederberg J.** 1950. The beta-d-galactosidase of *Escherichia coli*, strain K-12. J Bacteriol **60:**381-392.
9. **Holloway BW.** 1955. Genetic Recombination in *Pseudomonas aeruginosa*. J Gen Microbiol **13:**572-581.
10. **Anyan ME, Amiri A, Harvey CW, Tierra G, Morales-Soto N, Driscoll CM, Alber MS, Shrout JD.** 2014. Type IV pili interactions promote intercellular association and moderate swarming of *Pseudomonas aeruginosa*. Proc Nat Acad Sci USA **111:**18013-18018.
11. **Mattingly AE, Kamatkar NG, Morales-Soto N, Borlee BR, Shrout JD.** 2018. Multiple Environmental Factors Influence the Importance of the Phosphodiesterase DipA upon *Pseudomonas aeruginosa* Swarming. Appl Environ Microbiol **84**.
12. **Shrout JD, Chopp DL, Just CL, Hentzer M, Givskov M, Parsek MR.** 2006. The impact of quorum sensing and swarming motility on *Pseudomonas aeruginosa* biofilm formation is nutritionally conditional. Mol Microbiol **62:**1264-1277.
13. **Weaver AA, Jia J, Cutri AR, Madukoma CS, Vaerewyck CM, Bohn PW, Shrout JD.** 2024. Alkyl quinolones mediate heterogeneous colony biofilm architecture that improves community-level survival. Journal of Bacteriology **206**.
14. **Lequette Y, Greenberg EP.** 2005. Timing and localization of rhamnolipid synthesis gene expression in *Pseudomonas aeruginosa* biofilms. J Bacteriol **187:**37-44.
15. **Kamatkar NG, Shrout JD.** 2011. Surface Hardness Impairment of Quorum Sensing and Swarming for *Pseudomonas aeruginosa*. PLoS ONE **6:**e20888.
16. **Weaver AA, Hasan NA, Klaassen M, Karathia H, Colwell RR, Shrout JD.** 2019. Prosthetic joint infections present diverse and unique microbial communities using combined

whole-genome shotgun sequencing and culturing methods. J Med Microbiol **68:**1507-1516.

17. **Keogh D, Tay WH, Ho YY, Dale JL, Chen S, Umashankar S, Williams RBH, Chen SL, Dunny GM, Kline KA.** 2016. Enterococcal Metabolite Cues Facilitate Interspecies Niche Modulation and Polymicrobial Infection. Cell Host Microbe **20:**493-503.
18. **Choi K-H, Schweizer HP.** 2006. mini-Tn7 insertion in bacteria with single attTn7 sites: example *Pseudomonas aeruginosa*. Nat Protocols **1:**153-161.
19. **Hoang TT, Karkhoff-Schweizer RR, Kutchma AJ, Schweizer HP.** 1998. A broad-host-range Flp-*FRT* recombination system for site-specific excision of chromosomally-located DNA sequences: application for isolation of unmarked *Pseudomonas aeruginosa* mutants. Gene **212:**77-86.
